# Arachidonic acid is associated with dyslipidemia and cholesterol-related lipoprotein metabolism signatures

**DOI:** 10.3389/fcvm.2022.1075421

**Published:** 2022-12-05

**Authors:** Fan Li, Yu Wang, Huahui Yu, Xiaoqian Gao, Linyi Li, Haili Sun, Yanwen Qin

**Affiliations:** Key Laboratory of Remodeling-Related Cardiovascular Diseases, Ministry of Education, National Clinical Research Center for Cardiovascular Diseases, Beijing Anzhen Hospital, Capital Medical University, Beijing Institute of Heart, Lung and Blood Vessel Disease, Beijing, China

**Keywords:** metabolomics, arachidonic acid, cholesterol metabolism, apolipoprotein B, dyslipidemia

## Abstract

**Introduction:**

Abnormal lipoprotein metabolism is associated with a variety of diseases, cardiovascular disease in particular. Free fatty acids (FAs) and triglycerides (TGs) are the principal lipid species in adipocytes and are the major components of lipoproteins. However, in routine clinical laboratory testing, only the total plasma concentrations of FAs and TGs are typically measured.

**Methods:**

We collected 965 individuals with hyperlipidemia plasma and clinical characteristics; high-throughput metabolomics permits the accurate qualitative and quantitative assessment of a variety of specific FAs and TGs and their association with lipoproteins; through regression analysis, the correlation between multiple metabolites and routine measured lipid parameters was found. Mice were fed a diet containing AA, and the concentrations of TC and TG in the plasma of mice were detected by enzyme method, western blot and qRT-PCR detected the protein and mRNA levels of cholesterol synthesis and metabolism in mice.

**Result:**

Using LC-MS/MS identified eight free FA and 27 TG species in plasma samples, the plasma concentrations of free arachidonic acid (AA) and AA-enriched TG species were significantly associated with the plasma low-density lipoprotein-cholesterol, apolipoprotein B (ApoB), and total cholesterol (TC) concentrations after adjustment for age, sex, the use of lipid-lowering therapy, and body mass index. AA-rich diet significantly increased the plasma concentrations of TC and ApoB and the liver expression of ApoB protein and reduced the protein expression of ATP binding cassette subfamily G members 5 and 8 in mice.

**Discussion:**

In this study, it was clarified that the plasma concentrations of free AA- and AA-enriched TG species were significantly associated with the plasma low-density lipoprotein-cholesterol, ApoB, and TC concentrations in individuals with hyperlipidemia, and it was verified that AA could increase the plasma TC level in mice. Taken together, these findings suggest a potential role of AA in the regulation of plasma cholesterol and lipoprotein concentrations.

## Introduction

Dyslipidemia is an important public health problem that is increasing in incidence and prevalence worldwide and is a driving force behind the progression of cardiovascular disease (CVD). In general, dyslipidemia is characterized by high circulating concentrations of low-density lipoprotein-cholesterol (LDL-C), triglycerides (TGs), and apolipoprotein B (ApoB); and low circulating concentrations of high-density lipoprotein-cholesterol (HDL-C) and apolipoprotein A1 (ApoA1). Hypercholesterolemia is the most important type of dyslipidemia, causing 172 million deaths annually (1). In 1961, researchers involved in the Framingham Heart Study identified a high serum concentration of cholesterol-related lipoproteins, and specifically of LDL-C, to be a risk factor for coronary heart disease (2).

The treatment of dyslipidemia is an ever-growing type of pharmacotherapy that targets various proteins involved in the synthesis, transport, or metabolism of lipoproteins (3). Fatty acids (FAs) and TGs are the principal lipid species in adipocytes and the major lipid components of lipoproteins. These species vary in the lengths of their carbon chains and their level of saturation. Numerous lines of evidence indicate that high free FA and TG concentrations increase the risks of atherosclerotic cardiovascular disease and mortality (4). However, in routine clinical laboratory testing, the total serum FA and TG concentrations are typically measured. Such assessments lack sufficient specificity and sensitivity and only show significant increases in severe dyslipidemia (5). Therefore, the accurate and sensitive quantification of various FA and TG species might permit a more granular assessment of dyslipidemia and better targeting of interventions.

Metabolomic techniques have the potential to provide insights into disease mechanisms by assisting with the identification of the underlying metabolic pathways and potential biomarkers. Blood metabolomic studies have already provided critical insights in the pathogenesis of complex diseases, such as cardiovascular and metabolic diseases (6, 7). Importantly, metabonomics and its derivative lipomics have become powerful methods of studying the relationships between biochemistry and clinical phenotypes. However, to date, few metabolomics studies have been performed in humans that aimed to characterize the relationships of plasma FA or TG signatures with dyslipidemia-related biomarkers and therapeutic targets. Therefore, in the present study, we used FA and TG profiles and clinical laboratory lipid data to identify clinically relevant metabolomic variations, and then studied the significance of the key metabolomic signature for the regulation of lipid metabolism in an animal model.

## Materials and methods

### Participants

A total of 965 patients with hypercholesterolemia who attended Beijing Anzhen Hospital between March 2017 and June 2018 were enrolled. The inclusion criteria were: age > 18 years, hypercholesterolemia, and the use of standard lipid-lowering therapy. The exclusion criteria were: a current or previous condition of any body system other than hypercholesterolemia, including familial hypercholesterolemia, serious cardiovascular and cerebrovascular disease (such as myocardial infarction, stroke, and heart failure), respiratory disease, infectious disease, chronic kidney disease, pregnancy, and malignancy. The diagnostic criteria for hypertension were patients with mean systolic blood pressure (SBP) ≥ 140 mmHg or diastolic blood pressure (DBP) ≥ 90 mmHg (three consecutive measurements, with an interval of 5 min), or 24-h systolic blood pressure ≥ 130 mmHg or 24-h diastolic blood pressure ≥ 80 mmHg, or those who received antihypertensive drugs and had a clear history of hypertension (8). According to the American diabetes Association, the diagnostic criteria for diabetes: having symptoms of diabetes (polyuria, polydipsia and unexplained weight loss) and blood glucose concentration of two blood samples obtained at random ≥ 200 mg/dl (11.1 mmol/L), fasting glucose FGP ≥ 126 mg/dl (7.0 mmol/l) and blood glucose concentration ≥ 200 mg/dl (11.1 mmol/L) in 2 h after 75-g anhydrous glucose load (OGTT test) (9). According to the guidelines for prevention and treatment of dyslipidemia in Chinese adults (revised in 2016), the diagnostic criteria for hypercholesterolemia are: plasma TC ≥ 6.2mmol/L (240mg/dl), LDL-C ≥ 4.1mmol/L (160mg/dl). Written informed consent was obtained from all the participants. The study conformed with the principles of the Declaration of Helsinki and was approved by the Ethics Committee of Beijing Anzhen Hospital of the Capital University of Medical Sciences.

### Data collection and clinical laboratory testing

The demographic characteristics of the participants, including their age, sex, body mass index (BMI), and use of lipid-lowering therapy, were recorded for each participant. Fasting blood samples were obtained to measure the serum TC, TG, LDL-C, HDL-C, non-HDL, ApoB, and ApoA1 concentrations using an automatic biochemistry analyzer (AU 5400, Beckman Coulter, Brea, CA, USA).

### Sample preparation and liquid chromatography-mass spectrometry analysis

One hundred-microliter aliquots of plasma were mixed with 400-μl aliquots of ice-cold chloroform-methanol (2:1, v/v) containing a series of stable isotope-labeled internal standards (0.01 mg/ml stearic acid-18, 18, 18-d3, 0.005 mg/ml palmitic acid-d5, 0.012 mg/ml arachidonic acid (AA)-d8, and 0.005 mg/ml TG (17:1/17:1/17:1)-d5). The mixture was vortexed for 5 min at 4^°^C and centrifuged at 15,000 rpm for 15 min at 4^°^C. After precipitating the proteins, the lower organic phases were collected and placed into clean, dry tubes, then evaporated to dryness. The dried residues were stored at –80^°^C until further analyzed.

The lipidomes of each plasma sample were characterized as previously described. Briefly, the residues were reconstituted in 200 μl of chloroform: methanol (1:1, v/v) and diluted three-fold with isopropanol: acetonitrile: water (2:1:1, v/v/v). The metabolites were separated using a reverse-phase X-select CSH C18 column (2.1 × 100 mm, 2.5 μm; Waters Corp., Milford, MA, USA). The mobile phase consisted of a linear gradient system of phase A (10 mM ammonium acetate and 0.1% formic acid in 60% acetonitrile/40% water) and phase B (isopropanol: acetonitrile, 9:1, v/v): 0 min 60% A, 2 min 57% A, 12 min 40% A, 12.1 min 25% A, 18 min 1% A, 19 min 1% A, and 20 min 40% A. The sample injection volume was 10 μl. The column temperature was maintained at 40^°^C and the flow rate was 0.4 ml/min. Mass spectrometric analyses were performed on a Q-Exactive HF MS (Thermo Fisher Scientific, Waltham, MA, USA) using a data-dependent acquisition model. The spray voltage was 3.3 kV for positive ion mode and 2.5 kV for negative ion mode. Each acquisition cycle consisted of one survey scan at 60,000 resolutions from 150 to 1,200 m/z, followed by 10 MS/MS scans at 15,000 resolutions, using step-NCEs of 15, 30, and 45. The dynamic exclusion was set to 10 s, the sheath gas flow rate was 40 L/h, and the aux gas was 10 L/h. The probe heater temperature and capillary temperature were set at 300^°^C and 320^°^C, respectively.

### Animal study design

Seven-week-old male C57BL/6J mice were purchased from Beijing Huafukang Biotechnology Co., Ltd. (Beijing, China) and were housed in the specific pathogen-free laboratory animal facility of Beijing Anzhen Hospital, Capital Medical University. The animal study was approved by the Institutional Animal Care and Use Committee of Capital Medical University. The mice were housed at 27^°^C in a temperature and light/dark cycle-controlled environment with free access to water and either standard rodent chow diet or AA-containing diet (10) (1.5g/kg, Changzhou shuyishu’er company, Changzhou, China) for 4 weeks (*n* = 6 per group), during which period their food intake was recorded. At the end of this period, all the mice were euthanized, their body and liver masses were recorded, and their tissues were dissected, cut into small pieces, and stored at –80^°^C.

### Biochemical assays

Blood samples were collected from the mice after 4 h of fasting, then serum was extracted and the concentrations of TC, TG, and ApoB, and the activities of ALT and AST were measured enzymatically using kits from the Nanjing Jiancheng Bioengineering Institute.

Lipids were extracted from 20–30-mg samples of frozen liver by adding 0.18–0.27 mL aliquots of absolute ethanol (volume ratio 9:1) and homogenizing the tissue at 4^°^C at 2,500 rpm for 10 min. The supernatants were collected and the TC and TG contents were determined enzymatically using kits from the Nanjing Jiancheng Bioengineering Research Institute.

### Liver histology

Liver samples were fixed in 4% paraformaldehyde for at least 24 h, embedded in paraffin, and 5-μm sections were prepared and fixed on glass slides. The sections were then stained with hematoxylin and eosin (HE) and examined using a light microscope (Nikon, Tokyo, Japan). Other liver samples were snap-frozen, embedded in OCT compound, and 8-μm frozen sections were prepared and stained with oil red O.

### Western blotting

Twenty-milligram samples of liver tissue were lysed in RIPA buffer (Solarbio, Beijing, China)by ultrasonication, the lysates were centrifuged at 12,000 rpm for 20 min, the supernatants were collected, and the protein content was analyzed using western blot analysis. Proteins were separated on sodium dodecyl sulfate-polyacrylamide gel electrophoresis and then transferred onto PVDF membranes. The membranes were blocked using 5% skimmed milk and then sequentially incubated with appropriate primary and secondary antibodies. The primary antibodies used were an anti-HMGCR polyclonal antibody, an anti-LDLR polyclonal antibody, an anti-PCSK9 polyclonal antibody, an anti-ApoB polyclonal antibody, an anti-SREBP2 polyclonal antibody, an anti-ABCG5 polyclonal antibody, and an anti-ABCG8 polyclonal antibody (all from Cohesion Biosciences, London, UK).

### Real-time polymerase chain reaction

Real-time polymerase chain reaction was used to quantify specific mRNA expression. RNA was isolated from liver samples using Trizol (Life Technologies, Thermo Fisher Scientific, Waltham, MA, USA) and converted to cDNA using a reverse transcription kit purchased from Thermo Fisher Scientific (Waltham, MA, USA). RT-PCR was performed using the SYBR green method on a real-time PCR system. The relative abundance of each mRNA species was evaluated using the 2^–ΔΔCT^ method. The primer sequences used were as follows: LDLR, forward 5′-CTGTAGGGGTCTTTACGTGTTC-3′, reverse 5′-GTTTTCCTCGTCAGATTTGTCC-3′; HMGCR, forward 5′-GGTGCAAAGTTCCTTAGTGATG-3′, reverse 5′- GAATAGACACACCACGTTCATG-3′; PCSK9, forward 5′-AG CAGCCAGGTGGAGGTGTATC-3′, reverse 5′-CTTGCTCGC CTGTCTGTGGAAG-3′; ApoB, forward 5′-CTGGCTGCCTA TCTCTTGCTGATG-3′, reverse 5′-GTTCTTCACCTGCTCAC TCTGTTCC-3′; SREBP2, forward 5′-TTTTACTGAAGTAGA GCGGGTC-3′, reverse 5′-CATGCATGGCTCTACAGGTATA-3′; ABCG5, forward 5′-CATTGAAAGAGCACGATACCTG-3′, reverse 5′-AGATTCTGAACGAGACGCATAA-3′; and ABCG8, forward 5′-GGACAAATTTGGATAAATGGGC-3′, reverse 5′-AGATTCTGAACGAGACGCATAA-3′.

### Statistical analysis

The raw metabolomic data were analyzed using MS-DIAL software v3.6. Deconvolution, alignment, and data reduction were performed to provide a comprehensive data matrix, which was normalized to the signals associated with the internal standards. FAs and TGs were identified using the MS-DIAL software by comparing the exact molecular masses and fragments using the HMDB, METLIN, and LIPIDMAPS databases. The matched metabolites were further identified using their isotopic distributions, retention times and MS/MS fragmentation patterns *versus* authentic standards comprising an in-house metabolite library. The relationships of metabolites with clinical lipid data were assessed using multi-variable regression analyses and the sums of the standardized metabolite values (z-scores), weighted according to their corresponding β-coefficients in SPSS version 26 (IBM Corp., Armonk, NY, USA), and Spearman’s rank correlation coefficients were calculated using an online bioinformatics platform.^1^ The animal data are expressed as mean ± standard error of the mean (SEM) and were evaluated using analysis of two-tailed Student’s *t*-test in Prism software (GraphPad, San Diego, CA, USA). Statistical significance was set at *p* < 0.05. A latent network for the relationships between metabolites and proteins/genes was generated using Function Analysis, Connect Analysis, and Path Explorer in Ingenuity Pathway Analysis (Qiagen Inc., Hilden, Germany).

## Results

### Demographic and clinical characteristics of the participants

The characteristics of the 965 participants (mean age, 32.1 ± 3.6 years; 60.8% men) in the study are shown in Table 1. The study sample at the time of metabolite quantification had a near-normal mean BMI (24.93 ± 2.46 kg/m^2^), a relatively low prevalence of diabetes mellitus (13.7%), and a favorable lipid profile (LDL-C 2.51 ± 0.55 mmol/L, HDL-C 1.18 ± 0.35 mmol/L, TG 1.54 ± 0.65 mmol/L), but a high prevalence of hypertension (27.5%). Diabetes was diagnosed when the fasting blood glucose concentration of the participant was ≥ 126 mg/dl (7.0 mmol/L).

**TABLE 1:** Characteristics of the study participants (*n* = 965).

Ages	52.3 ± 12.8
Male gender (*n*,%)	587, 60.8%
Body mass index, kg/m^2^	24.93 ± 2.46
Hypertension	265, 27.5%
Diabetes mellitus	132, 13.7%
Fasting glucose, mmol/L	5.66 ± 0.79
Low-density lipoprotein cholesterol (LDL-C), mmol/L	2.51 ± 0.55
High-density lipoprotein cholesterol (HDL-C), mmol/L	1.18 ± 0.35
Total cholesterol (TC), mmol/L	4.18 ± 0.71
Triglycerides (TG), mmol/L	1.54 ± 0.65
non-HDL-C, mmol/L	2.99 ± 0.61
Apolipoprotein A1 (APOA1), g/L	1.18 ± 0.24
Apolipoprotein B (APOB), g/L	0.89 ± 0.16
Statin alone (*n*,%)	234, 24.2%
Statins + Ezetimibe (*n*,%)	731, 75.8%

### Relationships of fatty acid and triglyceride species with routinely measured circulating lipid concentrations

**FIGURE 1:**
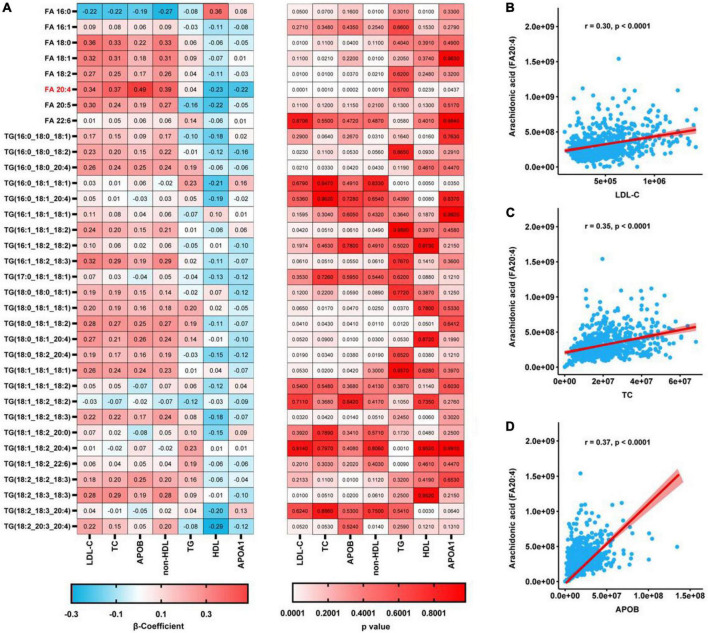
Regression analyses of the relationships between plasma metabolites and the concentrations of lipids conventionally measured in a clinical setting. **(A)** Relationships between each metabolite and conventionally measured lipid, after adjustment for age, sex, LLT, BMI, fasting glucose concentration, and the prevalences of hypertension and diabetes mellitus, determined using regression analyses. *P*-values were corrected using the Benjamini-Hochberg approach. **(B–D)** Spearman’s rank correlations for the relationships of AA with LDL-C **(B)**, TC **(C)**, and ApoB **(D)**.

**FIGURE 2:**
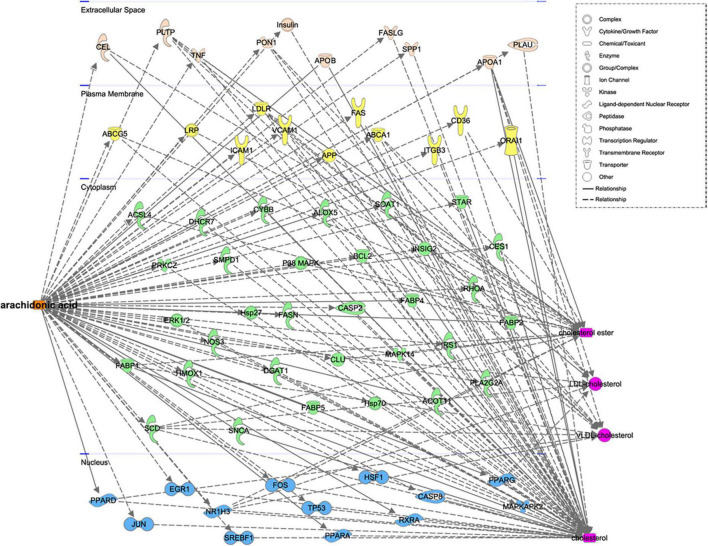
Functional network for the relationship between AA and related genes and proteins, constructed using Ingenuity pathway analysis.

We identified eight free FA and 27 TG species in plasma samples using LC-MS/MS, as shown in Figure 1A. We next analyzed the relationships between these metabolites with various routinely measured lipids and lipoproteins of interest in the participants. In regression analyses adjusted for the age, sex, LLT, BMI, fasting glucose concentration, and presence or absence of hypertension and diabetes mellitus of the participants, we found significant associations between multiple metabolites and routinely measured lipid parameters (Figure 1A). Notably, the concentrations of free AA (FA:20:4) and several arachidonic acyl TG species were positively associated with those of LDL-C, TC, ApoB, and non-HDL. Moreover, the AA concentration was negatively associated with those of HDL-C and ApoA1. Using Spearman’s rank correlation coefficients (Figures 1B–D), we found that the concentration of AA positively correlated with those of LDL-C (r = 0.30, *p* < 0.0001), TC (r = 0.35, *p* < 0.0001), and ApoB (r = 0.37, *p* < 0.0001). To further characterize the relationships between AA and the gene and proteins involved in lipoprotein metabolism, pathway analysis was performed. As shown in Figure 2, we generated a network indicating that AA is associated with multiple proteins and genes that regulate lipoprotein metabolism.

### Arachidonic acid consumption increases the plasma TC concentration in mice

**FIGURE 3:**
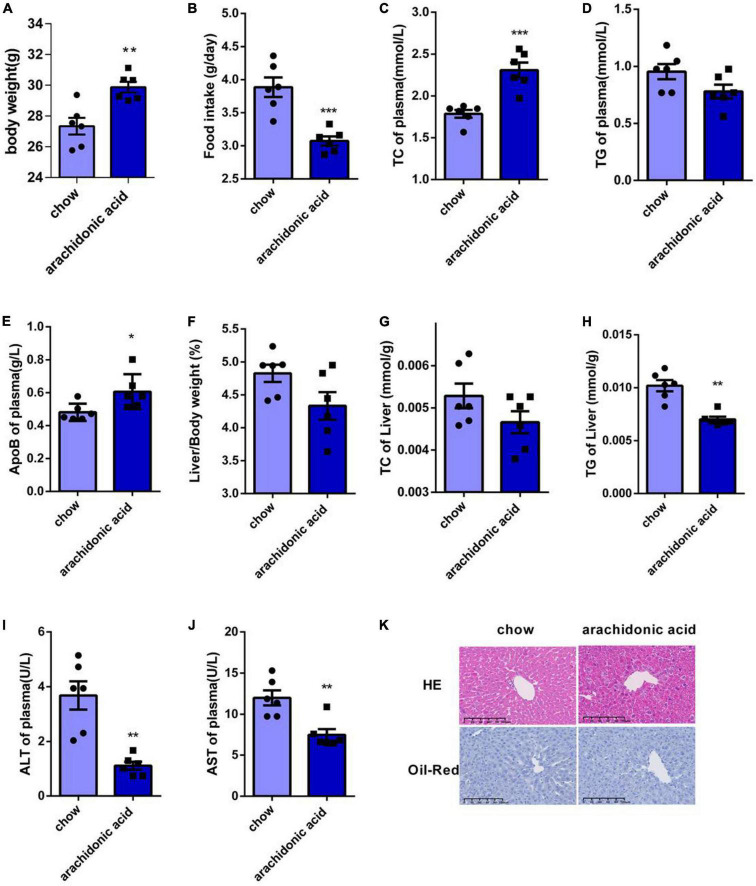
Arachidonic acid consumption increases the plasma total cholesterol concentrations of mice. Seven-week-old male mice (*n* = 6 in each group) were fed normal chow or a diet containing 1.5g/kg arachidonic acid for 4 weeks. **(A)** Body mass. **(B)** Food intake. **(C)** Plasma TC concentration. **(D)** Plasma TG concentration. **(E)** Plasma ApoB concentration. **(F)** Liver mass-to-body mass ratio. **(G)** Liver TC content. **(H)** Liver TG content. **(I)** Plasma alanine aminotransferase (ALT) activity. **(J)** Plasma aspartate aminotransferase (AST) activity. **(K)** Liver sections, stained with HE and oil red O. Data are expressed as mean ± SEM. The unpaired two-tailed Student’s *t*-test was used to compare the groups. *p < 0.05;**p < 0.01;***p < 0.0001 compared with the chow group.

We next studied the effects of AA on the plasma and liver lipid concentrations of mice. Mice were fed a diet containing AA (1.5 g/kg) for 4 weeks, and although the AA-fed mice were heavier than the chow-fed mice (Figure 3A), their food intake was lower (Figure 3B). The serum TC concentration of the mice fed the AA diet was higher than that of the chow-fed mice (Figure 3C), and although the serum TG concentrations did not differ (Figure 3D), the serum ApoB concentration was also significantly higher (Figure 3E).

Because the liver is the key organ for cholesterol metabolism, we weighed the livers of the mice and found no significant difference in the liver-to-body mass ratio of the two groups (Figure 3F). In addition, we measured the liver TC and TG contents of the mice, and found that the TC content did not differ (Figure 3G), but that the TG concentration was lower in the AA diet-fed mice (Figure 3H). However, the plasma serum AST and ALT activities of the AA-fed mice were lower than those of the chow-fed mice (Figures 3I–J). HE staining of sections showed damage to the livers of AA-fed mice (Figure 3K), and oil red O staining showed that there was no lipid deposition in the liver of these mice (Figure 3K). Thus, AA consumption causes an increase in plasma TC concentration in mice.

### Mechanism whereby AA consumption affects the plasma total cholesterol concentration

**FIGURE 4:**
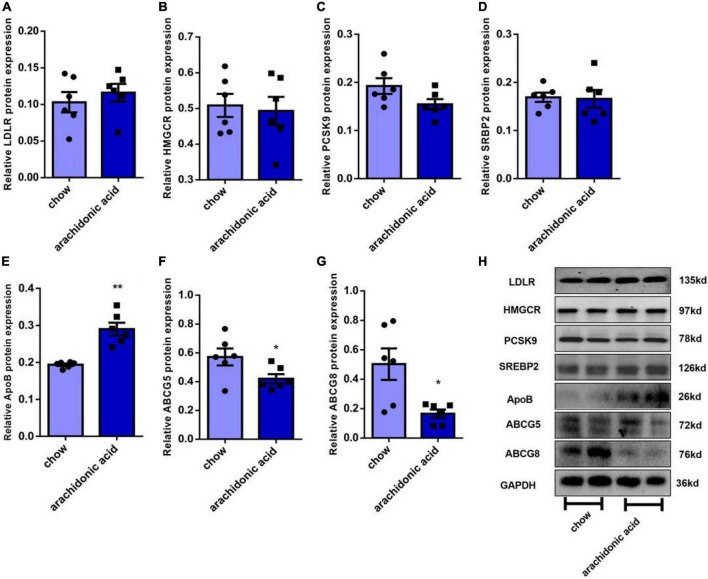
Effects of arachidonic acid on the expression of proteins involved in cholesterol-related pathways. Seven-week-old male mice (*n* = 6 in each group) were fed a chow diet or a diet containing 1.5g/kg arachidonic acid for 4 weeks. The expression of the following liver proteins was measured using western blot analysis: **(A)** LDLR, **(B)** HMGCR, **(C)** SREBP2, **(D)** PCSK9, **(E)** ApoB, **(F)** ABCG5, and **(G)** ABCG8. **(H)** Representative images of western blots. Data are expressed as mean ± SEM. The unpaired two-tailed Student’s *t*-test was used to compare the groups. *p < 0.05; **p < 0.01 compared with the chow group.

**FIGURE 5:**
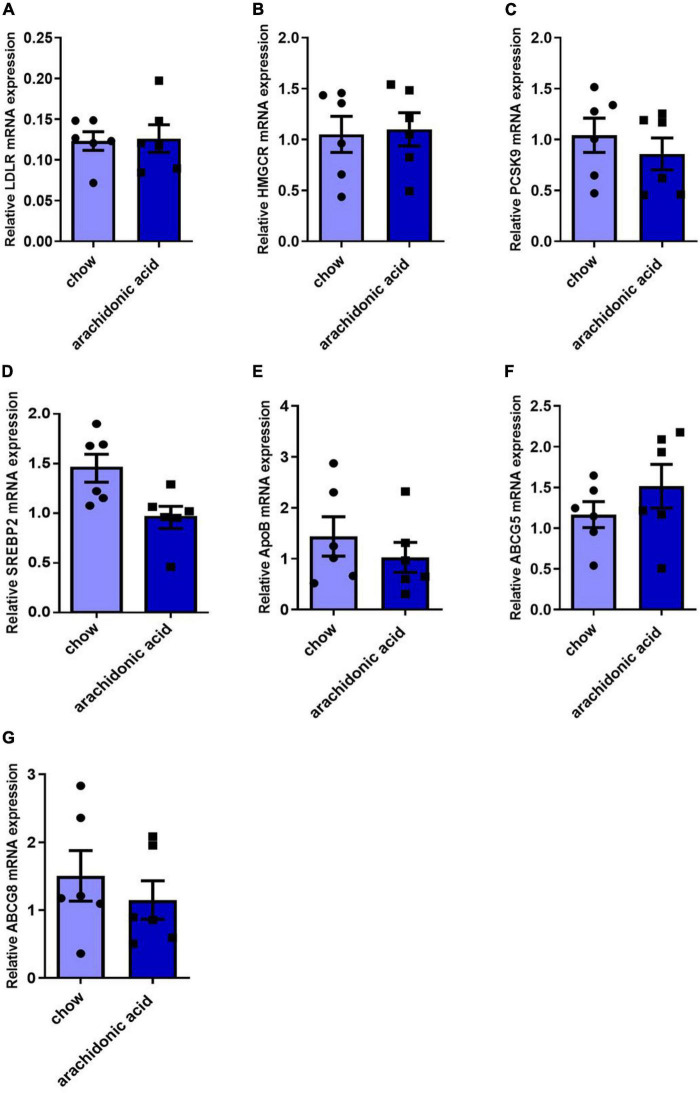
Effects of arachidonic acid on the mRNA expression of genes encoding proteins involved in cholesterol-related pathways. Seven-week-old male mice (*n* = 6 in each group) were fed a chow diet or a diet containing 1.5g/kg arachidonic acid for 4 weeks. The hepatic expression of the following mRNA species was measured using qRT-PCR: **(A)** *LDLR*, **(B)** *HMGCR*, **(C)** *PCSK9*, **(D)** *SREBP2*, **(E)** *ApoB*, **(F)** *ABCG5*, and **(G)** *ABCG8*. Data are expressed as mean ± SEM. The unpaired two-tailed Student’s *t*-test was used to compare the groups.

The liver is the key organ for cholesterol synthesis and metabolism. To investigate the mechanisms whereby AA consumption induces hypercholesterolemia in mice, liver lysates were prepared and western blotting analysis was performed to assess the protein expression of mediators of cholesterol synthesis and metabolism (LDLR, HMGCR, PCSK9, ABCG5, ABCG8, ApoB, and SREBP2) (Figures 4A–H). In addition, the hepatic mRNA expression of *LDLR, HMGCR, PCSK9, ABCG5, ABCG8, ApoB, and SREBP2* was measured (Figures 5A–G). LDLR is a main player responsible for the uptake of cholesterol by peripheral cells from the circulation. PCSK9 and ApoB regulate the absorption of cholesterol in circulating plasma. HMGCR and SREBP2 are primarily responsible for activation of genes involved in cholesterol synthesis. The ABCG5/ABCG8 heterodimer promotes cholesterol removal from the body (11). We found that the protein expression of ApoB (Figure 4E) was high and that of ABCG5 and ABCG8 (Figures 4F,G) was low in the livers of mice fed the AA diet. However, the protein expression of LDLR, HMGCR, PCSK9 and SREBP2 (Figures 4A–D) and the mRNA expression of LDLR, HMGCR, PCSK9, ABCG5, ABCG8, ApoB, and SREBP2 (Figure 5) did not differ between the groups. Therefore, we speculate that AA consumption may cause an increase in plasma TC concentration by increasing the hepatic ApoB expression and reducing that of ABCG5 and ABCG8.

## Discussion

In the present study, we used an untargeted metabolomic technique to measure the concentrations of trace lipids in the plasma of adults, and then analyzed the relationships of the various FAs and TGs with the concentrations of routinely measured plasma lipids. We found that the plasma FA 20:4 (arachidonic acid, AA) concentration closely correlated with those of TC, LDL-C, and ApoB, and therefore we hypothesized that AA affects cholesterol metabolism. To further explore the role of AA in cholesterol metabolism, we fed mice a diet containing 1.5 g/kg AA and found that this caused an increase in the plasma TC concentration of the mice.

The roles of lipids in health and disease is receiving a great deal of attention. Numerous previous studies have shown that lipids have multiple roles in atherosclerosis (12). Currently, the lipid types that are measured most frequently in a clinical setting include TC, TG, LDL-C, non-HDL-C, and ApoB. Lipids are heterogeneous substances, but FAs specifically play important roles (12). FAs are carboxylic acids with a very long carbon chains and a methyl group at one end and a carboxyl group at the other (13). FAs readily enter cells from the plasma, where they are either oxidized to produce energy in the form of ATP or re-esterified for storage as TGs (14). Both TGs and FAs are important constituents of lipoproteins. However, the relationships of the concentrations of particular TGs and FAs with those of TC, TG, LDL-C, and ApoB, which are conventionally measured in a clinical setting, have not been characterized.

In recent years, metabolite profiling has been performed using both targeted and untargeted metabolomics (5). In the present study, we used untargeted metabolomics to quantify the plasma concentrations of various FAs and TGs in the plasma of 965 adults and then evaluated their relationships with the plasma concentrations of TC, LDL-C, ApoB, non-HDL, TG, HDL, and ApoA1, measured using conventional techniques. The results revealed close correlations between the free fatty acid FA20:4 and the plasma concentrations of TC, LDL-C, ApoB, and non-HDL.

Arachidonic acid refers to a free FA containing 20 carbon atoms and 4 double bonds and is one of the most abundant polyunsaturated fatty acids in the human body (15) and has a variety of physiological functions, including as a component of the phospholipid bilayer of cell membranes, as a regulator of gene expression, as an inflammatory intermediate, and as a vasodilator/vasoconstrictor (15, 16). Previous studies have shown that AA-derived metabolites play important roles in the mechanisms of cardiovascular health and disease, in particular related to inflammation and atherosclerosis (17). Some circulating AA is converted to various epoxydiketones, and some is acylated to form TG (18). In human milk, AA is mainly present in the form of TG (19). TG stored in cytoplasmic lipid droplets is hydrolyzed by adipose triglyceride lipase, which releases AA (20). Drug use can affect the levels of AA in the body. For example, it has been reported that the amount of AA decreases following the treatment of hypercholesterolemia using simvastatin (21).

Arachidonic acid can be metabolized *via* three pathways that generate a wide range of derivatives with multiple functions. First, cyclooxygenases 1 (COX1) and 2 (COX2), also known as prostaglandin G/H synthases, generate thromboxane A2 (TXA2), prostacyclin (PGI2), and several prostaglandins (PGs) (22). Second, four lipoxygenases (LOXs), namely 5-LOX, 8-LOX, 12-LOX, and 15-LOX, metabolize AA. For example, 5-LOX generates 5-hydroperoxyeicosanoic acid (5-HPETE), 5-hydroxyeicosanoic acid (5-HETE), 5-oxy-ecosanoic acid (5-oxy-ete), and various leukotrienes (LTs) (23). Third, the cytochrome P450 (CYP450) pathway, which principally functions in the liver, is known for its role in detoxification, and includes cyclooxygenase and ω-hydroxylases, generates epoxyeicosapentaenoic acids (EETs) and hydroxyeicosapentaenoic acids (HETEs), respectively (15, 24). EETs have been reported to cause vasodilation, and this pathway and its metabolites are currently targeted for the treatment of CVD, including hypertension, heart failure, and stroke (25, 26). Numerous studies have demonstrated the role of 5-LOX-products in the pathogenesis and progression of CVD, and especially atherosclerosis, myocardial infarction, stroke, aortic aneurysm, and endothelial hyperplasia (26, 27). CYP-derived EET and ischemic cardiomyopathy: Ischemic myocarditis refers to CVD caused by insufficient oxygen supply to the heart (28). Hypercholesterolemia is principally caused by the accumulation of lipids in the body secondary to lipid metabolic disorders. However, it has not previously been reported whether AA affects tissue cholesterol concentrations.

Functional metabolomics is a useful approach to study the relationships between metabolites and diseases and the mechanisms involved. Untargeted metabolomics was used to identify succinate as a biomarker and therapeutic target for patients with aneurysms and coarctation and to determine the nature of the link (29). Functional metabolomics have also been used to identify a key role for Neu5Ac in acute myocardial infarction, which led to the suggestion that neuraminidase-1 may represent a therapeutic target for CAD (30). In the present study, we used a functional metabolomics approach to identify a link between AA and lipid metabolism, and then added this to normal mouse feed at a concentration of 1.5 g/kg and fed this to C57BL/6J mice for 4 weeks. At the end of this period, the mice were sacrificed and blood samples were collected. Analysis of these samples showed that the plasma TC concentration of AA-fed mice was higher than that of the chow-fed group, whereas the TG concentrations of the two groups were similar.

Cholesterol metabolism includes biosynthesis, absorption, output and esterification. Many proteins or genes are involved in cholesterol metabolism. LDLR is the primary receptor for cholesterol on peripheral cells and mediates its uptake from the circulation (11, 31), and is a transcriptional target of SREBP2. The endocytosis of LDL-derived cholesteryl esters is followed by metabolism through the endolysin system, eventually liberating cholesterol (11, 32). PCSK9 is one of the nine proprotein convertases of the secreted serine protease family and binds to LDLR (11, 32). ABCG5 and ABCG8 are principally expressed in the intestine and liver, where they form heterodimers and are essential for cholesterol absorption. Dietary cholesterol binds to ABCG5 and ABCG8 on the intestinal epithelium, which enter the intestinal lumen and aid the absorption process (11, 33). ApoB is the only carrier protein that characterizes LDL, and if it is mutated, a protein is produced that is unable to bind to LDLR (11). We prepared mouse liver lysates and measured the expression of proteins involved in cholesterol metabolism in mice fed the AA or chow diet. We found that the protein expression of ApoB in the liver of the AA group was higher and that of the ABCG5 and ABCG8 was lower than in mice fed the chow diet, which suggests that AA may regulate the plasma cholesterol concentration of mice through effects on ApoB, ABCG5, and ABCG8 expression.

In summary, we have shown a relationship of the plasma concentration of AA (FA20:4) with those of the conventionally measured lipids TC, LDL-C, and ApoB in humans. To further characterize the effect of AA on plasma cholesterol, we fed mice a diet containing 1.5 g/kg AA for 4 weeks, which increased their plasma TC concentration. We also found that AA consumption increased ApoB protein expression and decreased protein expression of ABCG5 and ABCG8 in mouse liver, which may mediate the effect. There are some limitations in this study, some unsolved questions need further exploration, such as whether different diets can change the level of AA and the comparison of AA levels in patients with hypercholesterolemia and healthy people.

## Data availability statement

The original contributions presented in this study are included in this article/supplementary material, further inquiries can be directed to the corresponding author.

## Ethics statement

The studies involving human participants were reviewed and approved by Ethics Committee of Beijing Anzhen Hospital of the Capital University of Medical Sciences. The patients/participants provided their written informed consent to participate in this study. The animal study was reviewed and approved by Animal Ethics Committee of Capital Medical University. Written informed consent was obtained from the individual(s) for the publication of any potentially identifiable images or data included in this article.

## Author contributions

YQ designed the experiments and managed the study. FL and YW performed the experiments and analyzed the data. HY, XG, LL, and HS collected blood samples and the clinical data. FL wrote the manuscript. All authors have read and approved the final version of the manuscript.

## Footnotes

1
http://www.bioinformatics.com.cn/login/
